# Increased anti-correlation between the left dorsolateral prefrontal cortex and the default mode network following Stanford Neuromodulation Therapy (SNT): analysis of a double-blinded, randomized, sham-controlled trial

**DOI:** 10.1038/s44184-024-00073-y

**Published:** 2024-07-06

**Authors:** Niharika Gajawelli, Andrew D. Geoly, Jean-Marie Batail, Xiaoqian Xiao, Adi Maron-Katz, Eleanor Cole, Azeezat Azeez, Ian H. Kratter, Manish Saggar, Nolan R. Williams

**Affiliations:** 1 Department of Psychiatry and Behavioral Sciences, Stanford University, Palo Alto, CA USA; 2 Neuropsychiatrie du comportement et du développement, Centre Hospitalier Guillaume Régnier, Université de Rennes, Rennes, France

**Keywords:** Depression, Neuroscience

## Abstract

SNT is a high-dose accelerated intermittent theta-burst stimulation (iTBS) protocol coupled with functional-connectivity-guided targeting that is an efficacious and rapid-acting therapy for treatment-resistant depression (TRD). We used resting-state functional MRI (fMRI) data from a double-blinded sham-controlled randomized controlled trial^1^ to reveal the neural correlates of SNT-based symptom improvement. Neurobehavioral data were acquired at baseline, post-treatment, and 1-month follow-up. Our primary analytic objective was to investigate changes in seed-based functional connectivity (FC) following SNT and hypothesized that FC changes between the treatment target and the sgACC, DMN, and CEN would ensue following active SNT but not sham. We also investigated the durability of post-treatment observed FC changes at a 1-month follow-up. Study participants included transcranial magnetic stimulation (TMS)-naive adults with a primary diagnosis of moderate-to-severe TRD. Fifty-four participants were screened, 32 were randomized, and 29 received active or sham SNT. An additional 5 participants were excluded due to imaging artifacts, resulting in 12 participants per group (Sham: 5F; SNT: 5F). Although we did not observe any significant group × time effects on the FC between the individualized stimulation target (L-DLPFC) and the CEN or sgACC, we report an increased magnitude of negative FC between the target site and the DMN post-treatment in the active as compared to sham SNT group. This change in FC was sustained at the 1-month follow-up. Further, the degree of change in FC was correlated with improvements in depressive symptoms. Our results provide initial evidence for the putative changes in the functional organization of the brain post-SNT.

## Introduction

Major depressive disorder (MDD) affects over 322 million people worldwide and is the leading cause of disability^2^. An estimated 30.9% of these patients have treatment-resistant depression (TRD), for whom standard antidepressant treatments are ineffective^3^. Repetitive transcranial magnetic stimulation (rTMS) of the left dorsolateral prefrontal cortex (L-DLPFC) is one treatment option in this setting. The L-DLPFC is negatively connected with the subgenual anterior cingulate cortex (sgACC)^4–6^, and stimulation of the area within the L-DLPFC most negatively connected to the sgACC is associated with greater antidepressant efficacy^4–6^. We recently reported a robust antidepressant effect in TRD of a high-dose, accelerated intermittent theta burst (a form of rTMS) protocol known as Stanford neuromodulation therapy (SNT) in a sham-controlled trial^1^. In SNT, resting-state fMRI (rsfMRI) data are used to target and stimulate the L-DLPFC area most negatively connected to the sgACC on an individual level. The intervention resulted in a 62% average reduction in depressive symptoms from baseline to post-treatment as measured by the Montgomery–Åsberg Depression Rating Scale (MADRS), with an effect size (Cohen’s *d*) of 1.7^1^. Antidepressant efficacy was primarily sustained through the 1-month follow-up. However, the neural basis underlying the antidepressant effect remains unclear.

Previous studies have suggested that FC differences within and between the default mode network (DMN) and the central executive network (CEN) may be relevant to the pathophysiology of depression and the mechanism of rTMS treatment. For example, FC within the DMN is higher in depressed individuals and decreases post-rTMS^7^. Similarly, the L-DLPFC becomes more negatively connected to the DMN post-rTMS in regions including the parahippocampal gyrus, medial prefrontal cortex (mPFC), and posterior cingulate cortex (PCC)^7^. As for the CEN, while no changes in FC post-rTMS in depression have been reported^7^, elevated functional connectivity density, a graph-based indicator of network organization, has been reported in the CEN following rTMS^8^.

To better understand the neural basis of SNT antidepressant effects, we explored FC changes in our recent double-blinded RCT^1^. Specifically, we employed a hypothesis-driven (ROI-based) analysis examining changes in FC between the L-DLPFC target and the sgACC, DMN (PCC, mPFC, bilateral temporal cortices)^9^, and CEN from baseline to immediately post and 1-month post-treatment. We also performed an exploratory (seed-to-whole-brain) analysis to identify voxel-wise changes in FC. Finally, we assessed correlations between FC changes and antidepressant effects.

## Methods

The trial was prospectively registered in the U.S. Clinical Trials registry (NCT03068715). All procedures were conducted in accordance with the ethical standards outlined in the Declaration of Helsinki. The study was approved by the Stanford University Institutional Review Board, and all participants provided written consent before participating in any study procedures.

### Clinical trial details

Treatment involved a 5-day high dose accelerated iTBS at the functional connectivity (fc)-guided L-DLPFC stimulation site. 182 TRD patients were screened online, 54 in person, and 32 participants who met the inclusion criteria were randomized in this double-blinded clinical trial. Two were excluded later for failing to meet the inclusion criteria, and one withdrew. Inclusion criteria were a primary diagnosis of treatment-resistant major depressive disorder, Hamilton Depression Rating Scale ≥20 and Montgomery and Åsberg Depression Rating Scale ≥ 20, and no prior TMS intervention^1^.

Of the remaining 29 participants, 14 received active SNT, and 15 received the sham protocol. Two participants were excluded from the analysis due to sessions with high in-scanner head motion (<80% EPI volumes with FD <0.5 mm). MADRS scores and MRI scans at baseline, post-treatment, and 1-month follow-up were collected. Three subjects were excluded from the follow-up data analysis due to missing data. This resulted in 12 participants in the active and sham groups investigated in this study.

The SNT RCT clinical analysis reported a response rate (reduction of ≥50% in MADRS score) of 71.4% immediately post-treatment^1^ in active participants. In the present analysis, with a smaller subset of the same cohort, we observed a response rate of 75% in active participants and 8.3% in sham, per MADRS, as well as the HAMD-17 (reduction of ≥50% in HAMD-17 score).

### Data acquisition

Structural and rsfMRI scans were acquired with a 3TGE Discovery MR750 scanner with a 32-channel head-neck imaging coil at the Center for Cognitive and Neurobiological Imaging at Stanford. GE’s “BRAVO” sequence (three-dimensional, T1-weighted) was used to collect high-resolution structural images for the whole brain (FOV = 256 × 256 mm; matrix = 256 × 256 voxels; slice thickness = 0.9 mm; TR = 2530 ms, TE = 2.98 ms, flip angle = 7°). The 8-min rsfMRI scan was collected with a simultaneous multi-slice acquisition echo planar imaging (EPI) sequence: TR = 2000 ms, TE = 30 ms, flip angle = 77°, slice acceleration factor = 3, FOV = 230 × 230 mm, matrix = 128 × 128 voxels, 1.8 × 1.8 mm^2^ in-plane resolution, 87 contiguous axial slices parallel to the anterior commissure–posterior commissure line. Participants were instructed to let their minds wander naturally and were shown a white fixation cross on a black screen.

### Data pre-processing

Anatomical MRI data were bias field corrected and processed using FreeSurfer. The fMRI data was preprocessed using the fmriprep version 20.2.5^10^, which included coregistration of the functional and anatomical images, spatiotemporal filtering, and resampling of the BOLD signal to standard spaces. 36 confounding variables, including six motion-related parameters, a global signal, a signal from the CSF, a white-matter signal, and their derivatives and derivatives-squared, were computed. The XCPEngine pipeline^11^ was later used to denoise fMRI data and estimate functional connectivity measures by generating nuisance time series to regress out the 36 confounding variables. Temporal censoring was performed using a framewise displacement threshold of >0.5 mm. Details of the process are described in Supplementary Methods.

### Data analysis

To examine the changes in FC associated with SNT, we first conducted an *ROI-based analysis* between the L-DLPFC target and predefined networks of interest and, consecutively, an L-DLPFC target *seed to whole-brain* convergence analysis. For both analyses, we first estimated a group × time (baseline vs. post-treatment) interaction while controlling for baseline covariates, followed by a within-group analysis (active) to examine whether any observed changes immediately post-treatment were sustained at 1 month. Based on previous reports^1,12–14^, we included age, sex, Maudsley Staging Method (MSM) score, and the time since MDD diagnosis as covariates for both ROI and whole-brain analyses. The MSM scores are included as they characterize treatment resistance in MDD, which could influence antidepressant response trajectories to iTBS^13^.

**Fig. 1:**
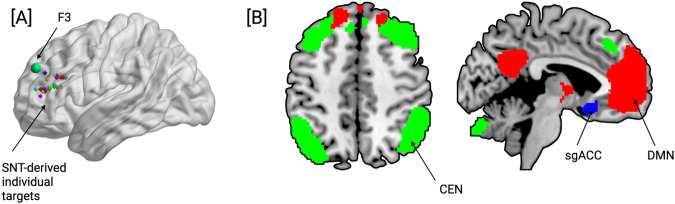
Personalized seeds and ROI. **A** Peaks of the 5 mm spherical seeds corresponding to the personalized L-DLPFC stimulation sites overlaid on a template brain. The larger green sphere is the average F3 location, often used in TMS targeting at MNI coordinates −35.5, 49.4,32.4. **B** Showing network-based and regional ROIs.

ROI-based connectivity analyses were conducted using a 5 mm spherical seed centered on the *personalized* L-DLPFC stimulation target. Figure 1A shows target centers overlaid on the brain and the standard F3 location^15^. We first examined FC changes between the L-DLPFC stimulation target and the predefined ROIs (sgACC, CEN, DMN), displayed in Fig. 1B. Baseline FC differences between the groups were also computed to evaluate potential pre-treatment differences. The sgACC ROI was defined using the Brodmann–Yale atlas (http://bioimagesuite.org). The DMN and CEN ROIs were defined from the FIND functional ROI atlase^16^, and the FC for these ROIs was calculated as the mean across voxels. To formally test the changes in FC between these ROIs and the L-DLPFC between the groups, we used repeated measures ANOVA in MATLAB (version 2022a, The MathWorks Inc., Natick, MA, USA).

The exploratory seed-to-whole-brain analysis uses the same L-DPLFC target as the seed for converging results across the analyses. The whole brain analysis was conducted on the fMRI Expert Analysis Tool (FEAT) on FSL 6.00^17,18^. The target time series was extracted for each individual, and the positive and negative correlations were computed in the lower-level analysis in the GLM model. Active and sham groups were then compared in the higher-level GLM model. Here, the *Z* (Gaussianised *T*/*F*) statistical images were thresholded non-parametrically using clusters determined by *Z* > 1.65 and a (corrected) cluster significance threshold of *P* = 0.05^19^. The aforementioned covariates were included in the group-level analysis. To visualize the direction of effect, we extracted the average functional connectivity values of the generated clusters for each participant.

## Results

### Baseline characteristics

Basic participant demographic information is presented in Table 1. No significant FC differences between groups existed at baseline [DMN: (*F*(1,18) = 0.016, *p* = 0.900), CEN:(*F*(1,18) = 0.429, *p* = 0.521), bilateral sgACC: (*F*(1,18) = 0.245, *p* = 0.626))], although we observed negative FC between the L-DLPFC target and the DMN at baseline for both active and sham groups (Supplementary Fig. 1).

**Table 1:** Demographics table at baseline

Variable		Sham (*n* = 12)	Active SNT (*n* = 12)	Test statistic	*p*
Age in years, mean (SD)		54.5 (16.5)	51.6 (14.2)	*t* = 0.454	0.386
Sex, no. of participants (%)				*χ*^2^ = 0	1
	Male	7 (29.2)	7 (29.2)		
	Female	5 (20.8)	5 (20.8)		
Maudsley staging method score		9.5 (1.7)	8.9 (1.7)	*t* = 0.852	0.865
Years since diagnosis		25.2 (14.6)	32.6 (17.1)	*t* = −1.14	0.676

### ROI and seed-based analyses

Our ROI-based FC analysis revealed a significant group x time interaction between the personalized L-DLPFC target and the DMN (*F*(1,18) = 4.717, *p* = 0.0435) but not for the bilateral sgACC (*F*(1,18) = 0.326, *p* = 0.575), or CEN ROIs *F*(1,18) = 0.175, *p* = 0.657). Since we were testing a priori hypotheses, we did not apply a correction for multiple comparisons. Post-hoc pairwise comparisons showed significant FC decreases from baseline to immediately post-treatment in the DMN for the active group (*p* = 0.011) but not the sham group (*p* = 0.569).

**Fig. 2:**
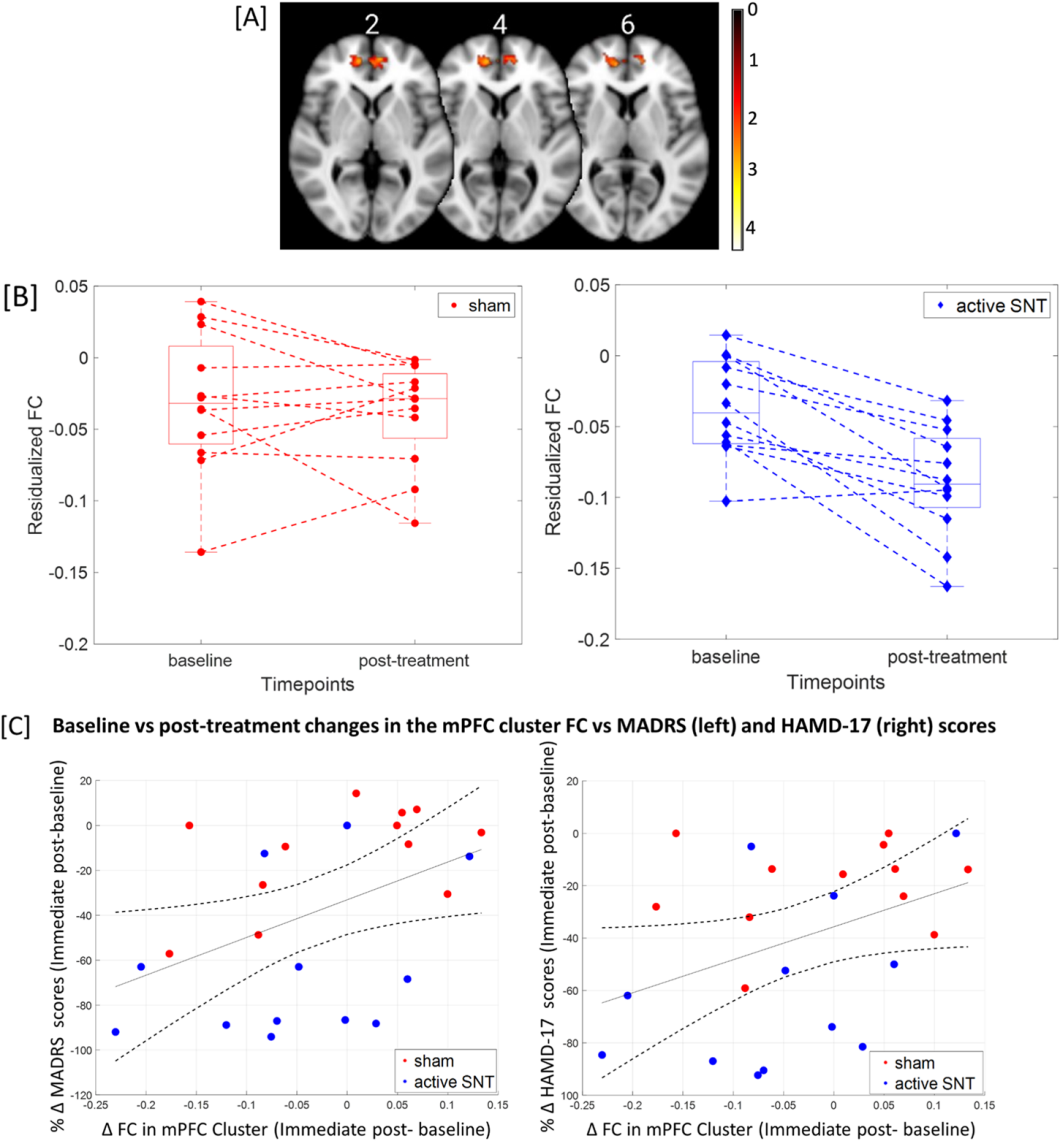
Results from the whole-brain seed-based analysis. **A** Medial prefrontal cortex cluster was detected through the whole-brain analysis with L-DLPFC seed, with a color bar indicating the intensity of the *z*-statistic. The images are labeled using the center slice as slice 0. **B** Combined box plot and individual residualized FC changes between L-DLPFC and the mPFC cluster shown in (**A**). The active group is displayed in blue, and the sham in red. **C** Scatter plots showing the correlations between FC changes in the mPFC and percentage changes of MADRS (left) and HAMD-17 (right) between baseline and post-treatment (*p* < 0.05 in both cases). The black line indicates linear fit through all data points, with dotted lines showing the 95% confidence interval.

Consistent with the ROI-based analysis, exploratory seed-to-whole-brain analysis also revealed a significant group × time interaction (*Z* > 1.65, *p* = 0.00496, cluster size = 729 voxels), such that FC decreased significantly from baseline to immediate post-treatment in a cluster within the canonical DMN region (mPFC) in the active group only (Fig. 2A and B).

To examine whether the observed changes in FC at post-treatment were sustained at the 1-month follow-up, we examined differences in FC between immediate post-treatment and 1-month follow-up visits within the active group. No significant differences were found for either the entire DMN in the ROI analysis or the mPFC cluster (*p* > 0.05) identified in the seed-whole brain analysis.

Finally, we examined whether changes in baseline to post-treatment FC between the L-DLPFC target and ROI-based results, as well as the L-DLPFC target to whole-brain results, correlated with improvement in depressive symptoms. We extracted FC changes for the ROI-based DMN and the corresponding seed-based mPFC cluster and computed Spearman’s partial correlations with the percentage change from baseline for the MADRS and HAMD-17 while accounting for age, sex, MSM, and years since diagnosis as covariates.

Scatter plots showing the relationship between clinical and seed-based FC changes are shown in Fig. 2C. No significant correlations between clinical improvement and FC changes in the ROI-based DMN were observed (*p* > 0.05). However, we did find significant correlations between FC changes in the seed-based mPFC cluster and the percent change in MADRS (rho = 0.4817, *p* = 0.031) and HAMD-17 (rho = 0.5033, *p* = 0.023) scores from baseline to immediately post-treatment.

## Discussion

FC-guided, targeted stimulation of the L-DLPFC in active participants resulted in significant changes in FC in DMN-associated ROIs post-treatment, providing the first evidence of an SNT-induced biological effect in a sham-controlled trial using a target-based FC approach. Interestingly, our ROI-based and whole-brain analyses converged at changes in FC between the target and mPFC (sub-region of DMN).

The L-DLPFC target site and DMN were negatively connected in most subjects at baseline (with or without covariates; see Supplementary Fig. 1) and without significant group differences. SNT led to an increased magnitude of negative connectivity in the active group following treatment, with a prominent negatively connected cluster in the mPFC. Such connectivity changes are consistent with prior studies of rTMS^7^. Further, we also observe a relation between L-DLPFC-mPFC FC changes and post-treatment clinical improvement. Similar relations were not observed in the previous works, possibly due to differences in treatment paradigm (5-week 10 Hz rTMS vs. 5-day aiTBS), L-DLPFC target seed definition (MNI coordinate vs. personalized FC-derived), baseline participant characteristics, concomitant medications, and image acquisition parameters. In our current study, we also observed that the FC between the target site and the DMN did not differ significantly in the active group between immediate-post and 1-month follow-ups, suggesting a likely sustained effect.

Recent work by Elbau et al.^20^ has prompted considerable debate surrounding FC between the L-DLPFC and the sgACC in personalized targeting approaches, finding that the effect of baseline FC was significant but weak, explaining about 3% of the variance in clinical outcomes. However, it is important to consider Elbau’s work within the framework of the THREE-D trial. Specifically, the authors examined the effects of baseline FC only in the context of open-label traditional daily treatment with 10 Hz rTMS, which led to remission in <30% of patients over 6 weeks. As such, the limited variability in remission rates may contribute, at least in part, to challenges in predicting individual variability. Moreover, concerning FC between the L-DLPFC and the sgACC, the BOLD fMRI spatial resolution (5 mm isotropic) in the THREE-D trial differs notably from the present study (1.8 mm isotropic). More importantly, perhaps, the mechanisms underlying 10 Hz rTMS and iTBS may not be identical, and the participation of the sgACC in their therapeutic effects could be indirect and temporally sensitive.

Targeting of the L-DLPFC in the present study is predicated on identifying clusters that are maximally negatively connected to the sgACC, for which there remains an abundance of evidence supporting baseline target-sgACC functional connectivity as a strong predictor of antidepressant response^21^. As such, we were particularly interested in whether a novel form of accelerated, excitatory (aiTBS) stimulation delivered to the personalized target might elicit subsequent target-sgACC functional connectivity changes, in contrast to previous studies utilizing traditional 10 Hz rTMS^7^. Interestingly, we found no significant FC changes between the L-DLPFC and the sgACC, which did not support our primary hypothesis. Still, our findings are at least partially in line with results previously reported by Liston et al., where 10 Hz rTMS was shown to enhance negative FC between the L-DLPFC and the medial prefrontal regions of the DMN but did not alter FC within the CEN or sgACC^7^.

In their recent sham-controlled trial examining in-vivo effects of a single session of iTBS to personalized L-DLPFC targets (based on sgACC FC) with concurrent iTBS-fMRI, Singh et al.^22^ demonstrated rapid changes in the connectivity profiles of the rACC and mPFC with respect to the DMN and Salience Network (SN). Interestingly, the evolution of downstream network connectivity coincided with an acute monotonic shift from negative to positive target-sgACC FC following iTBS. This mechanistic picture differs from their previous work^23^, which demonstrated a transient increase followed by a sustained decrease in target-sgACC FC coinciding temporally with DLPFC-DMN and sgACC-DMN FC changes post-10 Hz rTMS. Their work highlights two important points relevant to the present study. The first is that the mechanistic effects of 10 Hz rTMS and iTBS may indeed be differentiable, and direct comparisons of results across treatment modalities should be made with caution. Secondly, both studies by Singh et al.,^22,23^ demonstrated rapid post-stimulation changes in L-DLPFC target-sgACC FC coinciding with DMN connectivity changes (albeit in distinguished patterns). This suggests an early but possibly indirect and/or transient participation of the sgACC, especially in the case of iTBS. Consequently, the L-DLPFC target-sgACC connection may serve as a relevant byway, permitting access to modulate DMN connectivity during acute stimulation, but accurately measuring the participation of the target-sgACC connection in large-scale network effects might require more acute methods, such as concurrent iTBS/rTMS-fMRI or in-session heart-rate variability^24^.

Importantly, our work may advance understanding of the biological mechanisms underlying the antidepressant effect of SNT following a full course of treatment. Specifically, we observed that improvement in depressive symptom burden was significantly correlated with enhancement of negative functional connectivity between the target site and the mPFC after treatment. We speculate that excitatory stimulation to the personalized L-DLPFC target enhances baseline negative FC between these regions and also results in subsequent modulation of the connectivity profile of the mPFC, normalizing the DMN hyper-connectivity present in MDD. A previous report^7^ proposed a similar model whereby rTMS to the L-DLPFC may attenuate hyperconnectivity within medial prefrontal regions of the DMN in patients with MDD. As such, future work should examine this question specifically by evaluating downstream mPFC FC changes following SNT to the L-DLPFC target site.

Our modest sample size of 12 participants per group represents a primary limitation of our work. Due to the resulting limited statistical power, we first looked at the group × time interaction from baseline to immediate-post treatment, and, where significant, we conducted additional exploratory within-group analyses comparing the immediate-post and 1-month follow-up. Putative functional connectivity changes that may have occurred between the immediate post-treatment visit and the 1-month follow-up visit (e.g., late-responders) are unlikely to be captured by this approach. However, most active participants in our analyzed subset (75%) met responder status immediately post-treatment, mitigating this concern.

Additionally, as previously discussed^1^ the high level of education, the proportion of males relative to females, and the presence of co-morbidities in our cohort limits the generalizability of our results to other populations. Moreover, the definition of personalized L-DLPFC target sites by way of constructing standardized spheres of 5 mm radius may not fully characterize the focality or depth of the TMS-induced electrical field. As such, future work should leverage non-invasive neuromodulation simulation tools for biologically realistic modeling of treatment-induced electrical fields and subsequent treatment target definition. Similarly, we cannot exclude the possibility that our results might differ due to alternative image processing approaches or improvements in functional imaging acquisition quality, especially concerning subcortical regions like the sgACC, which are prone to signal dropout. However, our procedure was consistent with commonly used FC analysis practices (see Supplementary Methods).

Finally, it is important to acknowledge that in our exploratory seed-based analysis, the relatively low statistical threshold of non-parametrically identifying significant clusters determined by *Z* > 1.65^25^ reflects challenges with statistical power in a small sample size and, thus, future work is needed to replicate these findings with larger sample sizes.

We investigated brain FC changes associated with a novel, accelerated, FC-guided form of iTBS known as SNT from a recent sham-controlled RCT of patients with TRD. Between the baseline and post-treatment time points, we found significant FC changes from the target seed to the DMN through an ROI-based analysis and to the mPFC through a seed-to-whole-brain connectivity analysis. These FC changes were sustained at the 1-month follow-up. We also showed that the degree of FC change was correlated with the percent improvement in depression symptoms. Further work examining FC throughout and following the treatment course will help to identify how FC profiles evolve over the course of treatment and whether FC changes precede, follow, or coincide with clinical improvements.

### Supplementary information

Supplementary information

## Data Availability

Due to the sensitivity of psychiatric patient data, our institutional review board requires individualized review before data sharing. We have produced anonymized imaging and behavioral data linked to the present findings for sharing with all scientists with research plans and data safeguarding plans complying with Stanford University guidelines. Please contact the corresponding author with data-sharing requests.

## References

[1] Cole EJ (2022). Stanford neuromodulation therapy (SNT): a double-blind randomized controlled trial. Am. J. Psychiatry.

[2] Friedrich MJ (2017). Depression is the leading cause of disability around the world. JAMA.

[3] Zhdanava, M. et al. The prevalence and national burden of treatment-resistant depression and major depressive disorder in the United States. *J. Clin. Psychiatry* **82**, 2 (2021). 10.4088/JCP.20m13699 33989464

[4] Cash RFH (2021). Using brain imaging to improve spatial targeting of transcranial magnetic stimulation for depression. Biol. Psychiatry.

[5] Weigand A (2018). Prospective validation that subgenual connectivity predicts antidepressant efficacy of transcranial magnetic stimulation sites. Biol. Psychiatry.

[6] Fox MD, Buckner RL, White MP, Greicius MD, Pascual-Leone A (2012). Efficacy of transcranial magnetic stimulation targets for depression is related to intrinsic functional connectivity with the subgenual cingulate. Biol. Psychiatry.

[7] Liston C (2014). Default mode network mechanisms of transcranial magnetic stimulation in depression. Biol. Psychiatry.

[8] Zheng A (2020). Two-week rTMS-induced neuroimaging changes measured with fMRI in depression. J. Affect. Disord..

[9] Hamilton JP, Farmer M, Fogelman P, Gotlib IH (2015). Depressive rumination, the default-mode network, and the dark matter of clinical neuroscience. Biol. Psychiatry.

[10] Esteban O (2019). fMRIPrep: a robust preprocessing pipeline for functional MRI. Nat. Methods.

[11] Ciric R (2018). Mitigating head motion artifact in functional connectivity MRI. Nat. Protoc..

[12] Loo CK (2018). International randomized-controlled trial of transcranial direct current stimulation in depression. Brain Stimul..

[13] Li C-T (2014). Efficacy of prefrontal theta-burst stimulation in refractory depression: a randomized sham-controlled study. Brain.

[14] Cole EJ (2020). Stanford accelerated intelligent neuromodulation therapy for treatment-resistant depression. Am. J. Psychiatry.

[15] Okamoto M (2004). Three-dimensional probabilistic anatomical cranio-cerebral correlation via the international 10–20 system oriented for transcranial functional brain mapping. Neuroimage.

[16] Shirer WR, Ryali S, Rykhlevskaia E, Menon V, Greicius MD (2012). Decoding subject-driven cognitive states with whole-brain connectivity patterns. Cereb. Cortex.

[17] Woolrich MW, Ripley BD, Brady M, Smith SM (2001). Temporal autocorrelation in univariate linear modeling of FMRI data. Neuroimage.

[18] Woolrich MW, Behrens TEJ, Beckmann CF, Jenkinson M, Smith SM (2004). Multilevel linear modelling for FMRI group analysis using Bayesian inference. Neuroimage.

[19] Worsley KJ (2001). Statistical analysis of activation images. Funct. MRI.

[20] Elbau IG (2023). Functional connectivity mapping for rTMS target selection in depression. Am. J. Psychiatry.

[21] Tura A, Goya-Maldonado R (2023). Brain connectivity in major depressive disorder: a precision component of treatment modalities?. Transl. Psychiatry.

[22] Singh A (2020). Default mode network alterations after intermittent theta burst stimulation in healthy subjects. Transl. Psychiatry.

[23] Singh A (2019). Personalized repetitive transcranial magnetic stimulation temporarily alters default mode network in healthy subjects. Sci. Rep..

[24] Dijkstra, E. et al. Transcranial magnetic stimulation-induced heart-brain coupling: implications for site selection and frontal thresholding—preliminary findings. *Biol. Psychiatry Global Open Sci.*10.1016/j.bpsgos.2023.01.003 (2023). 10.1016/j.bpsgos.2023.01.003 PMC10593873 37881544

[25] Eklund A, Nichols TE, Knutsson H (2016). Cluster failure: why fMRI inferences for spatial extent have inflated false-positive rates. Proc. Natl Acad. Sci. USA.

