# Supplementary material for: Increased anti-correlation between the left dorsolateral prefrontal cortex and the default mode network following Stanford Neuromodulation Therapy (SNT): analysis of a double-blinded, randomized, sham-controlled trial
Source: Npj Ment Health Res. 2024 Jul 6;3:35. doi: 10.1038/s44184-024-00073-y (PMC11227523; doi:10.1038/s44184-024-00073-y)
Supplement: Supplementary file 1 — Supplementary information [file 44184_2024_73_MOESM1_ESM.docx]

Increased anti-correlation between the left dorsolateral prefrontal cortex and the default mode network following Stanford Neuromodulation Therapy (SNT): analysis of a double-blinded, randomized, sham-controlled trial

Niharika Gajawelli ¶, Andrew D. Geoly ¶, Jean-Marie Batail, Xiaoqian Xiao, Adi Maron-Katz, Eleanor Cole, Azeezat Azeez, Ian H. Kratter, Manish Saggar*, Nolan R. Williams*

Department of Psychiatry and Behavioral Sciences,

Stanford University

**Supplementary Methods**

*SNT Protocol*

Active or sham SNT protocols were randomly assigned to the participants. The treatments were administered with a MagVenture MagPro X100 system (MagVenture A/S Denmark) with a double-sided MagVenture Cool-B65 A/P coil. The TMS coil was positioned over each individual’s stimulation target site using a localite neuronavigation system (Localite GmbH, Sankt Augustin, Germany). The individualized stimulation sites varied in depth. Therefore, depth-corrected intensities were used. Stimulation was adjusted for the target depth and applied at 90% of the resting motor threshold (rMT). The stimulation intensity never exceeded 120% of rMT. Participants were administered 10 sessions of active or sham iTBS daily (18000 pulses/day) for 5 consecutive days (90000 pulses). Refer to previous publications regarding the treatment for more details^4^**.**

*Anatomical data preprocessing*

A total of 3 T1-weighted (T1w) images were found within the input BIDS dataset. All of them were corrected for intensity non-uniformity (INU) with N4BiasFieldCorrection[^26^](https://paperpile.com/c/RG9QUm/K9FHO), distributed with ANTs 2.3.3 [^26^](https://paperpile.com/c/RG9QUm/K9FHO). The T1w-reference was then skull-stripped with a *Nipype* implementation of the antsBrainExtraction.sh workflow (from ANTs), using OASIS30ANTs as the target template. Brain tissue segmentation of cerebrospinal fluid (CSF), white matter (WM) and gray matter (GM) was performed on the brain-extracted T1w using fast (FSL 5.0.9 [^27^](https://paperpile.com/c/RG9QUm/COSGE). A T1w-reference map was computed after registration of 3 T1w images (after INU-correction) using mri_robust_template (FreeSurfer 6.0.1[^28^](https://paperpile.com/c/RG9QUm/8lM2S)). Brain surfaces were reconstructed using recon-all (FreeSurfer 6.0.1[^29^](https://paperpile.com/c/RG9QUm/GTsX4)), and the brain mask estimated previously was refined with a custom variation of the method to reconcile ANTs-derived and FreeSurfer-derived segmentations of the cortical gray-matter of Mindboggle[^30^](https://paperpile.com/c/RG9QUm/Hymwp). Volume-based spatial normalization to two standard spaces (MNI152NLin6Asym, MNI152NLin2009cAsym) was performed through nonlinear registration with antsRegistration (ANTs 2.3.3), using brain-extracted versions of both T1w reference and the T1w template. The following templates were selected for spatial normalization: *FSL’s MNI ICBM 152 non-linear 6th Generation Asymmetric Average Brain Stereotaxic Registration Model* [^31^](https://paperpile.com/c/RG9QUm/giyNx), *ICBM 152 Nonlinear Asymmetrical template version 2009c* [^32^](https://paperpile.com/c/RG9QUm/geUVc).

*Functional data preprocessing*

*FMRIPrep Pipeline:* For each of the BOLD runs found per subject (across all tasks and sessions), the following preprocessing was performed. First, a reference volume and its skull-stripped version were generated using a custom methodology of *fMRIPrep*. Susceptibility distortion correction (SDC) was omitted. The BOLD reference was then co-registered to the T1w reference using bbregister (FreeSurfer), which implements boundary-based registration[^33^](https://paperpile.com/c/RG9QUm/Rg7kS). Co-registration was configured with six degrees of freedom. Head-motion parameters with respect to the BOLD reference (transformation matrices and six corresponding rotation and translation parameters) are estimated before any spatiotemporal filtering using mcflirt (FSL 5.0.9[^34^](https://paperpile.com/c/RG9QUm/k4b1W). The BOLD time-series were resampled onto the following surfaces (FreeSurfer reconstruction nomenclature): *fsaverage5*, *fsaverage*. The BOLD time-series (including slice-timing correction when applied) were resampled onto their original, native space by applying the transforms to correct for head-motion. These resampled BOLD time-series will be called *preprocessed BOLD in original space* or just *preprocessed BOLD*. The BOLD time-series were resampled into several standard spaces, correspondingly generating the following *spatially-normalized, preprocessed BOLD runs*: MNI152NLin6Asym, MNI152NLin2009cAsym. First, a reference volume and its skull-stripped version were generated using a custom methodology of *fMRIPrep*. FD and DVARS are calculated for each functional run, both using their implementations in *Nipype* (following the definitions by Power et al. 2014). The three global signals are extracted within the CSF, the WM, and the whole-brain masks. Results included in this manuscript come from preprocessing performed using *fMRIPrep* 20.2.5[^11,35^](https://paperpile.com/c/RG9QUm/7CXyn+rZZoD), which is based on *Nipype* 1.6.1[^36,37^](https://paperpile.com/c/RG9QUm/JhQWH+z1fYU).

*XCPEngine Pipeline*

The XCPEngine[^12^](https://paperpile.com/c/RG9QUm/B4rUM) pipeline was used to denoise fMRI data and estimate functional connectivity measures. The pipeline involves generating nuisance time series to regress out confounding variables. Our data was processed with 36 nuisance variables, which included 6 motion parameters (3 translational and 3 rotation), mean WM and CSF time series, and global signal regression (totaling 9 parameters), as well as the temporal derivatives of these 9 parameters, and squares of the original and temporal derivatives of the 9 parameters (totaling 18). Temporal censoring was also done using the Framewise Displacement rate calculated in fMRIPrep, where data points with high displacement (>0.5 mm) were removed from the data. The pipeline also applied temporal filtering with a low-pass cutoff of 0.08Hz and a high-pass cutoff of 0.01Hz, allowing the frequencies within this range. The data were spatially smoothed using the FSL SUSAN kernel with FWHM=6mm, reducing the spurious artifacts that may arise from noise from physiological signals or scanner activity.

**Supplementary Figures**

**
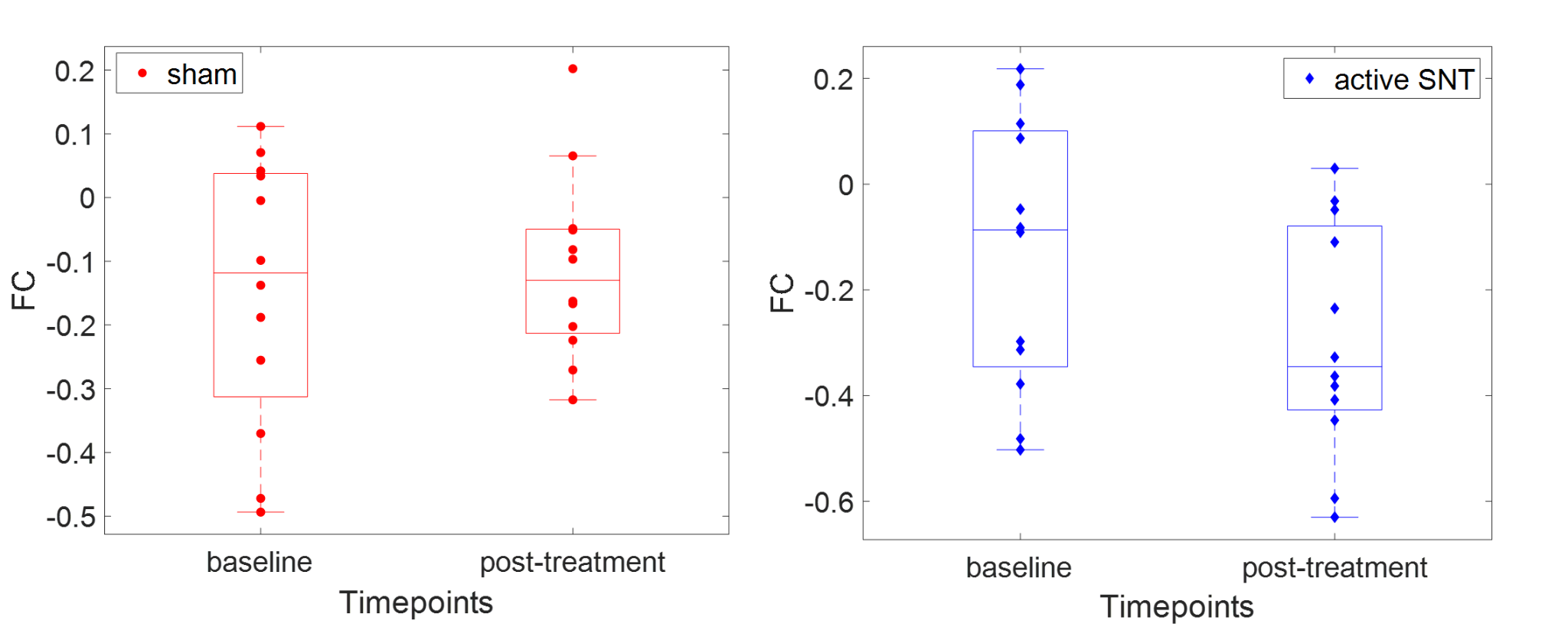
**

**Supplementary Figure 1**. DMN FC differences between baseline and post-treatment between the sham and active groups without covariates. We see the active group has reduced FC post-treatment compared to the sham group, even in the absence of covariates.


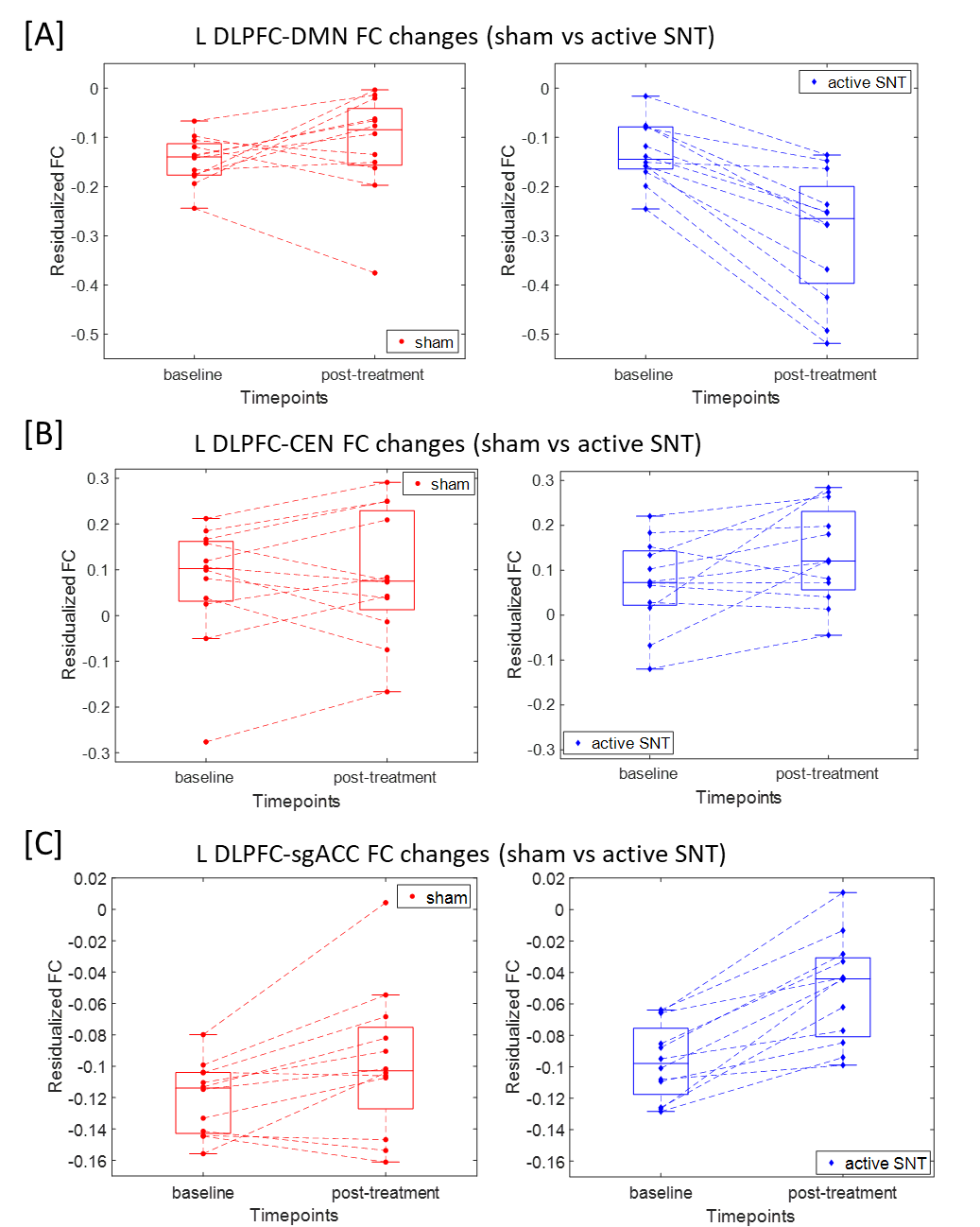


**Supplementary Figure 2.** [A] L-DLPFC and DMN FC differences between baseline and post-treatment for the sham and active groups (significant). [B] L-DLPFC and CEN FC differences between baseline and post-treatment for the sham and active groups (non-significant). [C] L-DLPFC and bilateral sgACC FC differences between baseline and post-treatment for the sham and active groups (non-significant).

**Supplementary References**

1. [Cole, E. J. *et al.* Stanford Neuromodulation Therapy (SNT): A Double-Blind Randomized Controlled Trial. *Am. J. Psychiatry* **179**, 132–141 (2022).](http://paperpile.com/b/RG9QUm/WZ5RY)

2. [Friedrich, M. J. Depression Is the Leading Cause of Disability Around the World. *JAMA* **317**, 1517 (2017).](http://paperpile.com/b/RG9QUm/0amUo)

3. [Zhdanava, M. *et al.* The Prevalence and National Burden of Treatment-Resistant Depression and Major Depressive Disorder in the United States. *J. Clin. Psychiatry* **82**, (2021).](http://paperpile.com/b/RG9QUm/k0ZuS)

4. [Cash, R. F. H. *et al.* Using Brain Imaging to Improve Spatial Targeting of Transcranial Magnetic Stimulation for Depression. *Biol. Psychiatry* **90**, 689–700 (2021).](http://paperpile.com/b/RG9QUm/fiMFV)

5. [Weigand, A. *et al.* Prospective Validation That Subgenual Connectivity Predicts Antidepressant Efficacy of Transcranial Magnetic Stimulation Sites. *Biol. Psychiatry* **84**, 28–37 (2018).](http://paperpile.com/b/RG9QUm/KLqZI)

6. [Fox, M. D., Buckner, R. L., White, M. P., Greicius, M. D. & Pascual-Leone, A. Efficacy of transcranial magnetic stimulation targets for depression is related to intrinsic functional connectivity with the subgenual cingulate. *Biol. Psychiatry* **72**, 595–603 (2012).](http://paperpile.com/b/RG9QUm/OMz9u)

7. [Liston, C. *et al.* Default mode network mechanisms of transcranial magnetic stimulation in depression. *Biol. Psychiatry* **76**, 517–526 (2014).](http://paperpile.com/b/RG9QUm/bymY2)

8. [Zheng, A. *et al.* Two-week rTMS-induced neuroimaging changes measured with fMRI in depression. *J. Affect. Disord.* **270**, 15–21 (2020).](http://paperpile.com/b/RG9QUm/eS6zV)

9. [Hamilton, J. P., Farmer, M., Fogelman, P. & Gotlib, I. H. Depressive Rumination, the Default-Mode Network, and the Dark Matter of Clinical Neuroscience. *Biol. Psychiatry* **78**, 224–230 (2015).](http://paperpile.com/b/RG9QUm/HDK54)

10. [Esteban, O. *et al.* poldracklab/fmriprep: 1.0.0-rc5. Preprint at https://doi.org/](http://paperpile.com/b/RG9QUm/BYjVb)[10.5281/zenodo.996169](http://dx.doi.org/10.5281/zenodo.996169) [(2017).](http://paperpile.com/b/RG9QUm/BYjVb)

11. [Esteban, O. *et al.* fMRIPrep: a robust preprocessing pipeline for functional MRI. *Nat. Methods* **16**, 111–116 (2019).](http://paperpile.com/b/RG9QUm/7CXyn)

12. [Ciric, R. *et al.* Mitigating head motion artifact in functional connectivity MRI. *Nat. Protoc.* **13**, 2801–2826 (2018).](http://paperpile.com/b/RG9QUm/B4rUM)

13. [Loo, C. K. *et al.* International randomized-controlled trial of transcranial Direct Current Stimulation in depression. *Brain Stimul.* **11**, 125–133 (2018).](http://paperpile.com/b/RG9QUm/TNI8i)

14. [Li, C.-T. *et al.* Efficacy of prefrontal theta-burst stimulation in refractory depression: a randomized sham-controlled study. *Brain* **137**, 2088–2098 (2014).](http://paperpile.com/b/RG9QUm/bE6bI)

15. [Cole, E. J. *et al.* Stanford Accelerated Intelligent Neuromodulation Therapy for Treatment-Resistant Depression. *Am. J. Psychiatry* **177**, 716–726 (2020).](http://paperpile.com/b/RG9QUm/i2pS8)

16. [Okamoto, M. *et al.* Three-dimensional probabilistic anatomical cranio-cerebral correlation via the international 10-20 system oriented for transcranial functional brain mapping. *Neuroimage* **21**, 99–111 (2004).](http://paperpile.com/b/RG9QUm/RDa2Y)

17. [Shirer, W. R., Ryali, S., Rykhlevskaia, E., Menon, V. & Greicius, M. D. Decoding subject-driven cognitive states with whole-brain connectivity patterns. *Cereb. Cortex* **22**, 158–165 (2012).](http://paperpile.com/b/RG9QUm/uqGJ6)

18. [Woolrich, M. W., Ripley, B. D., Brady, M. & Smith, S. M. Temporal autocorrelation in univariate linear modeling of FMRI data. *Neuroimage* **14**, 1370–1386 (2001).](http://paperpile.com/b/RG9QUm/2pMfY)

19. [Woolrich, M. W., Behrens, T. E. J., Beckmann, C. F., Jenkinson, M. & Smith, S. M. Multilevel linear modelling for FMRI group analysis using Bayesian inference. *Neuroimage* **21**, 1732–1747 (2004).](http://paperpile.com/b/RG9QUm/4qMB5)

20. [Worsley, K. J. Statistical analysis of activation images. *Functional MRI: An introduction to methods* **14**, 251–270 (2001).](http://paperpile.com/b/RG9QUm/7gbnd)

21. [Elbau, I. G. *et al.* Functional Connectivity Mapping for rTMS Target Selection in Depression. *Am. J. Psychiatry* **180**, 230–240 (2023).](http://paperpile.com/b/RG9QUm/JAf6l)

22. [Tura, A. & Goya-Maldonado, R. Brain connectivity in major depressive disorder: a precision component of treatment modalities? *Transl. Psychiatry* **13**, 196 (2023).](http://paperpile.com/b/RG9QUm/S4XM)

23. [Singh, A. *et al.* Default mode network alterations after intermittent theta burst stimulation in healthy subjects. *Transl. Psychiatry* **10**, 75 (2020).](http://paperpile.com/b/RG9QUm/oDyq3)

24. [Singh, A. *et al.* Personalized repetitive transcranial magnetic stimulation temporarily alters default mode network in healthy subjects. *Sci. Rep.* **9**, 5631 (2019).](http://paperpile.com/b/RG9QUm/6ObZg)

25. [Dijkstra, E. *et al.* Transcranial Magnetic Stimulation–Induced Heart-Brain Coupling: Implications for Site Selection and Frontal Thresholding—Preliminary Findings. *Biological Psychiatry Global Open Science* (2023) doi:](http://paperpile.com/b/RG9QUm/TeXfe)[10.1016/j.bpsgos.2023.01.003](http://dx.doi.org/10.1016/j.bpsgos.2023.01.003)[.](http://paperpile.com/b/RG9QUm/TeXfe)

26. [Tustison, N. J. *et al.* N4ITK: improved N3 bias correction. *IEEE Trans. Med. Imaging* **29**, 1310–1320 (2010).](http://paperpile.com/b/RG9QUm/K9FHO)

27. [Zhang, Y., Brady, M. & Smith, S. Segmentation of brain MR images through a hidden Markov random field model and the expectation-maximization algorithm. *IEEE Trans. Med. Imaging* **20**, 45–57 (2001).](http://paperpile.com/b/RG9QUm/COSGE)

28. [Reuter, M., Rosas, H. D. & Fischl, B. Highly accurate inverse consistent registration: a robust approach. *Neuroimage* **53**, 1181–1196 (2010).](http://paperpile.com/b/RG9QUm/8lM2S)

29. [Dale, A. M., Fischl, B. & Sereno, M. I. Cortical surface-based analysis. I. Segmentation and surface reconstruction. *Neuroimage* **9**, 179–194 (1999).](http://paperpile.com/b/RG9QUm/GTsX4)

30. [Klein, A. *et al.* Mindboggling morphometry of human brains. *PLoS Comput. Biol.* **13**, e1005350 (2017).](http://paperpile.com/b/RG9QUm/Hymwp)

31. [Evans, A. C., Janke, A. L., Collins, D. L. & Baillet, S. Brain templates and atlases. *Neuroimage* **62**, 911–922 (2012).](http://paperpile.com/b/RG9QUm/giyNx)

32. [Fonov, V. S., Evans, A. C., McKinstry, R. C., Almli, C. R. & Collins, D. L. Unbiased nonlinear average age-appropriate brain templates from birth to adulthood. *Neuroimage* **47**, S102 (2009).](http://paperpile.com/b/RG9QUm/geUVc)

33. [Greve, D. N. & Fischl, B. Accurate and robust brain image alignment using boundary-based registration. *Neuroimage* **48**, 63–72 (2009).](http://paperpile.com/b/RG9QUm/Rg7kS)

34. [Jenkinson, M., Bannister, P., Brady, M. & Smith, S. Improved Optimization for the Robust and Accurate Linear Registration and Motion Correction of Brain Images. *Neuroimage* **17**, 825–841 (2002).](http://paperpile.com/b/RG9QUm/k4b1W)

35. [Esteban, O. *et al.* fmriprep. *Softw. Pract. Exp.* (2018).](http://paperpile.com/b/RG9QUm/rZZoD)

36. [Gorgolewski, K. J., Esteban, O., Markiewicz, C. J. & Ziegler, E. Nipype. *Softw. Pract. Exp.* (2018).](http://paperpile.com/b/RG9QUm/JhQWH)

37. [Gorgolewski, K. *et al.* Nipype: a flexible, lightweight and extensible neuroimaging data processing framework in python. *Front. Neuroinform.* **5**, 13 (2011).](http://paperpile.com/b/RG9QUm/z1fYU)
